# Dandy Walker-like malformation in an adult cat with seizures: clinical description and MRI characteristics

**DOI:** 10.1177/20551169231174199

**Published:** 2023-06-23

**Authors:** Sara Formoso, Hannah Padley, Lisa Alves

**Affiliations:** 1 Department of Small Animal Medicine, Queen’s Veterinary School Hospital, University of Cambridge, Cambridge, UK; 2 Anderson Moores Veterinary Specialists, Hursley, Hampshire, UK

**Keywords:** Feline epilepsy, cerebellum, vermis, CNS, hypoplasia, ataxia, forebrain

## Abstract

**Case summary:**

A 2-year-old male neutered domestic shorthair cat was referred for investigation of a 10-month history of self-limiting, generalised tonic–clonic seizures. The cat was reported to be normal interictally but had always had a static abnormal gait. General physical examination was unremarkable. Neuroanatomical localisation was compatible with a diffuse cerebellar and diffuse forebrain lesion. Complete blood count, biochemistry, bile acid stimulation test, urinalysis, cisternal cerebrospinal fluid (CSF) analysis, *Toxoplasma gondii* serology and *T gondii* polymerase chain reaction in CSF were all unremarkable. MRI revealed an abnormal caudal fossa, absent cerebellar vermis and small cerebellar hemisphere with distension of the fourth ventricle. There were no forebrain abnormalities identified in the MRI or CSF changes that could justify the seizures. Considering the clinical presentation, the cat’s neurological examination and MRI features, a presumptive diagnosis of Dandy Walker-like malformation (DWLM) and epilepsy of unknown aetiology was made.

**Relevance and novel information:**

This is the first case report of an adult cat diagnosed with cerebellar malformation resembling DWLM and concomitant seizures, its MRI characteristics and long-term follow-up. The 3-year follow-up consultation revealed static neurological status with 2–4 seizures per year. The cat’s quality of life remained good at the time of writing.

## Case description

A 2-year-old male neutered domestic shorthair cat was referred for investigation of a 10-month history of self-limiting, generalised tonic–clonic seizures. The epileptic seizures were associated with loss of consciousness, tonic-clonic involuntary movement of all four limbs and autonomic signs (urination and ptyalism). The seizures occurred at night, and lasted less than 2 mins with no prodromal or postictal signs. The cat was reported to be interictally normal.

Phenobarbital (phenoleptil; Dechra) at 1.4 mg/kg PO q12h was prescribed by the referring veterinarian 7 months before presentation. Serial phenobarbital serum levels carried out at the referring veterinary practice were on the lower reference interval (RI) (45–50 µmol/l; RI 40–160 µmol/l). The cat remained seizure free for 6 months. However, 3 weeks before presentation, the cat had eight further similar seizures. Complete blood count, full biochemistry and bile acid stimulation test performed at the referring veterinarian were unremarkable. Phenobarbital serum levels remained at 50 µmol/l and the dose of phenobarbital (phenoleptil; Dechra) was increased to 2.4 mg/kg q12h 2 weeks before presentation.

The cat had been in the owners’ possession since he was 9 months old. According to the owners and to the rescue centre, the cat’s gait had always been abnormal. The cat was able to ambulate a few steps before falling to either side but it did not appear in pain. The cat was kept indoors with padded floors and walls. Since the cat’s gait had remained stable, the owners never investigated the origin of the abnormal gait. The cat’s vaccinations were up to date, and it ate a complete feline commercial diet.

On presentation, 3 weeks after the last seizure, the general physical examination was unremarkable. Interictally neurological examination revealed appropriate mentation with normal behaviour, and a generalised broad base stance. The cat was ambulatory with a marked cerebellar ataxia comprising dysmetria of the head, neck and all limbs, generalised kinesigenic hypertonicity and falling to both sides. On postural reactions, paw replacing was normal but had delay hopping in all four limbs with an exaggerated dysmetric response. Visual and tactile placing were delayed on the left thoracic and pelvic limbs. Segmental spinal reflexes were normal and the muscle tone was increased in all limbs. Cranial nerves were all normal except for absent bilateral menace responses with perceived normal vision. Neuroanatomical localisation was compatible with a diffuse cerebellar lesion based on neurological examination and diffuse forebrain lesion due to the reported seizure activity.

Urinalysis, including culture via cystocentesis, cisternal cerebrospinal fluid (CSF) analysis, including total protein, total nucleated cell count and cytology, *Toxoplasma gondii* serology and *T gondii* polymerase chain reaction in CSF were all unremarkable. A repeated phenobarbital serum level was 81 µmol/l (RI 40–160 µmol/l).

**Figure 1:**
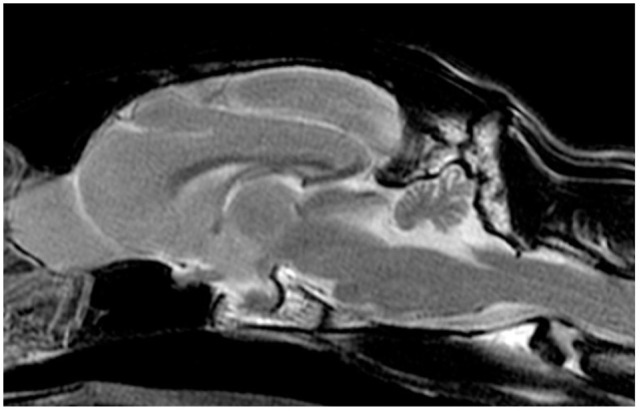
Sagittal T2-weighted (T2W) MRI at the level of the inter thalamic adhesion. Note the hypoplastic vermis, abnormal caudal fossa, misshaped occipital bone and distension of the fourth ventricle

**Figure 2:**
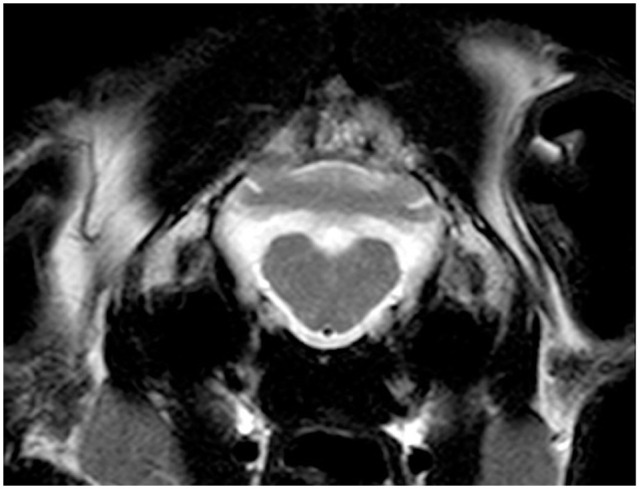
Transverse T2-weighted (T2W) MRI at the level of the cerebellar nuclei. Note the hypoplastic vermis and misshapen cerebellar hemispheres

**Figure 3:**
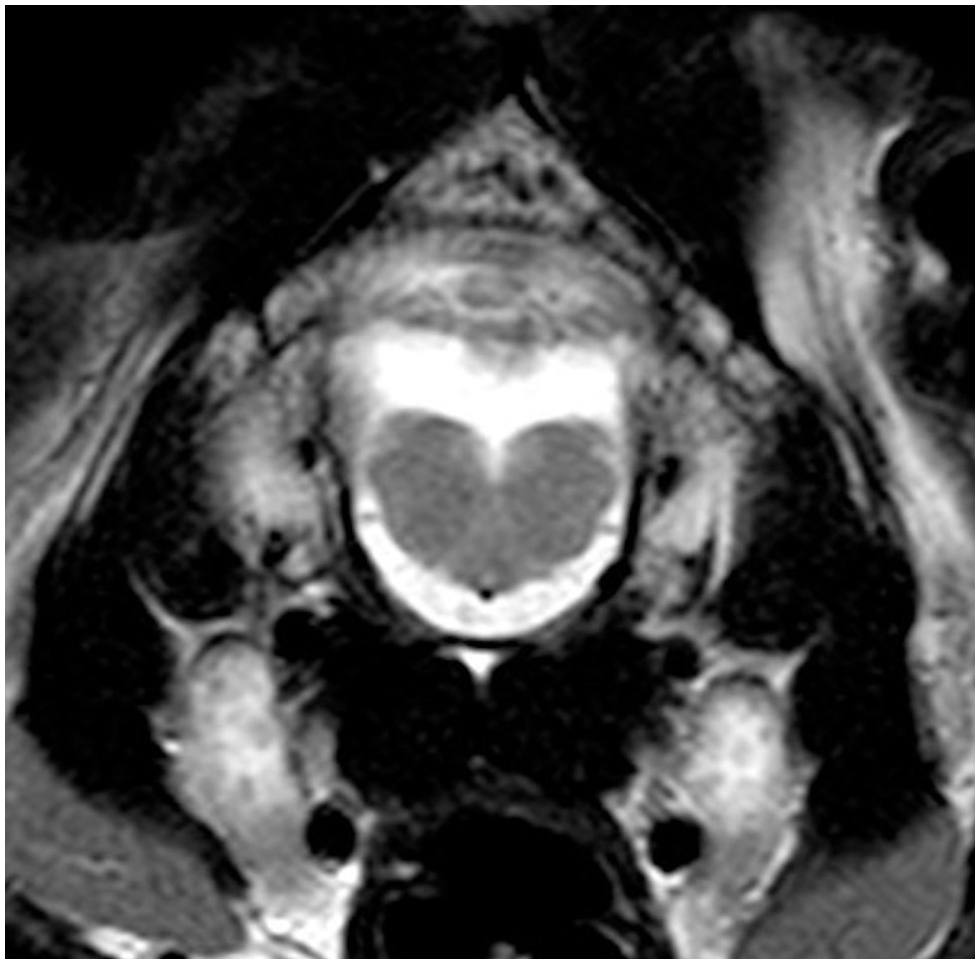
Transverse T2-weighted (T2W) MRI at the level of the caudal cerebellum and medulla oblongata. Note the aplastic caudal vermis

**Figure 4:**
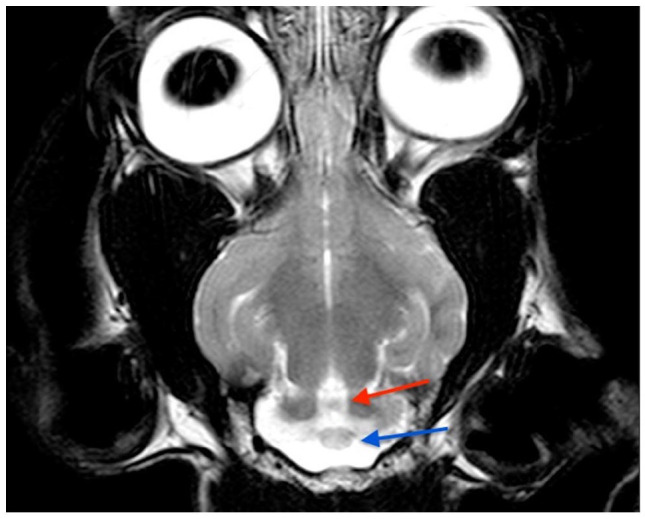
Dorsal T2-weighted (T2W) MRI at the level of the colliculi. Visible lingula (red arrow) and small uvula (blue arrow)

The cat underwent brain MRI, using a 1.5-T scanner (Phillips Intera 1.5 T system; Philips Medical Systems), under general anaesthesia in sternal recumbency. The following sequences were acquired: sagittal, transverse and dorsal T2-weighted (T2W) planes; sagittal, transverse and dorsal T1-weighted (T1W); transverse T2W fluid-attenuated inversion recovery (FLAIR) and transverse T2*-weighted gradient echo (T2*W). After intra-venous administration of a gadolinium-based contrast medium (Gadovist 1.0 mmol/ml; Bayer) at a dose of 0.1 mmol/kg, T1W sagittal, transverse and dorsal sequences were repeated. The caudal fossa of the calvarium was small and misshapen, with a pointed dorsal occipital bone. The cerebellum was markedly malformed; the cerebellar vermis was hypoplastic and more severe in the caudal region. Cerebellar hemispheres were small, irregularly shaped and appeared tethered dorsally to the calvarium. There was an abnormal small uvula and lingula, and the ansiform and paramedian lobules were not clearly defined (Figures 1–4).

**Figure 5:**
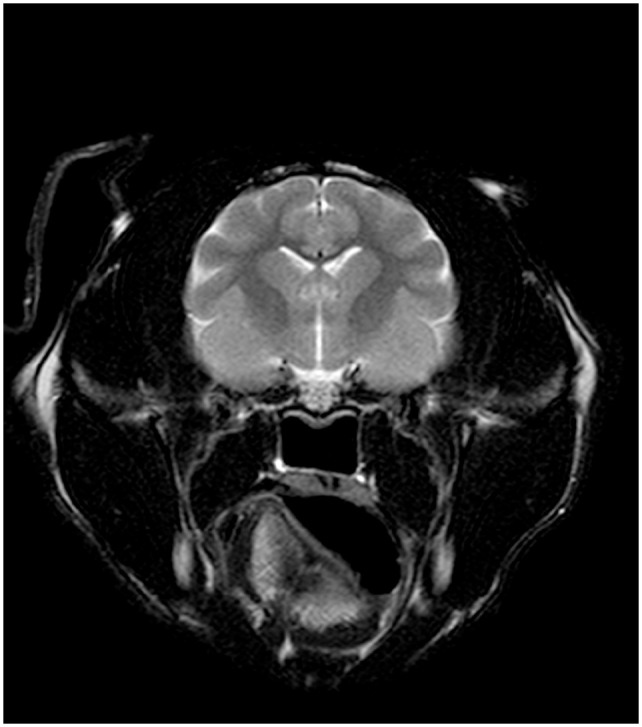
Transverse T2-weighted (T2W) MRI at the level of the caudate nucleus showing normal forebrain of the cat

Surrounding the small and misshapen cerebellum, there was an excess of T2W hyperintense and T1W/FLAIR hypointense fluid consistent with CSF in an enlarged fourth ventricle. The mesencephalic aqueduct was caudally dilated. The rest of the brain MRI and the postcontrast study did not reveal any further abnormalities (Figure 5).

Considering the clinical presentation, the cat’s neurological examination and MRI features, a presumptive diagnosis of Dandy Walker-like malformation (DWLM) and epilepsy of unknown aetiology was made. There were no forebrain abnormalities identified on the MRI or CSF changes that could justify the seizures. Phenobarbital 2.4 mg/kg (phenoleptil; Dechra) PO q12h was continued. At a 3-year follow-up consultation via telephone, the seizure frequency was stable (3–4 seizures per year), the gait remained unchanged and the owners perceived the cat as having a good quality of life.

## Discussion

Dandy Walker malformation (DWM) is a congenital abnormality described in people and in animals, and is characterised by agenesis or hypoplasia of the cerebellar vermis, cystic dilation of the fourth ventricle and enlarged posterior fossa with displacement of the tentorium and torcula.^1–5^

In animals, an additional focal or generalised hypoplasia of the cerebellar hemispheres has been reported.^5^ DWLM has therefore been suggested as the appropriate terminology in veterinary medicine.^5^

DWLM is a rare disease and to date the only reported feline case is on a kitten diagnosed through CT.^3^ However, the best diagnostic imaging modality to study the caudal fossa^6^ and the cerebellum anatomy is MRI.

This novel case describes the presence of cerebellar malformation resembling DWLM with concomitant seizures in an adult cat, its MRI features, clinical characteristics and long-term follow-up.

The main MRI findings in our cat consisted of hypoplasia of most of the cerebellar vermis and of both cerebellar hemispheres, cystic-like distension of the fourth ventricle and an abnormal shaped caudal fossa with dorsal occipital bone malformation. The remaining MRI showed a normal forebrain and brainstem. These MRI characteristics resembled DWLM as described in dogs.^5^

In veterinary patients, the most common reported clinical sign in DWLM is a non-progressive cerebellar ataxia associated with rolling, head tremors, abnormal postural reactions and reduced menace responses.^4,5,7–11^ This is in accordance with our case, as the cat’s main neurological deficits were severe static cerebellar ataxia and bilateral absent menace responses, as expected with a cerebellar neuroanatomical localisation. The cat’s limb movements were erratic and spastic, with a delay in protraction and once the movement was initiated, it was sudden and over-exaggerated. This lack of motor planning and delayed onset of movement can be seen with lesions affecting the cerebellar hemispheres. The absence of coordination of movements of the head and trunk and the changes noted in the cat’s muscle tone can be explained by the hypoplastic vermis.^12^

Interestingly, and despite the severe gait dysfunction, the main presenting complaint of our cat was seizure activity. Seizures are rarely reported in people diagnosed with DWM.^13–15^ DWM plus seizures are normally associated with additional findings, such as hypertensive hydrocephalus and corpus callosum malformation. Despite this, the presence of seizures without forebrain pathology has been reported in people diagnosed with DWM,^13,15^ although the pathophysiology remains unknown. In animals, seizures in association with DWLM have been reported in four dogs. Most of these dogs had additional forebrain malformations described on MRI, including hydrocephalus.^5^

In the present case, the MRI could not identify any macroscopic structural abnormalities in the forebrain to explain the seizures. However, it is possible that subtle cortical malformations, such as cortical heterotopia or dysplasia, were missed. In addition, brain inflammation was also less likely as there was no abnormal contrast enhancements on the MRI study and the CSF analysis was unremarkable. Metabolic and toxic causes were also deemed unlikely based on normal haematological, biochemical investigation, bile acid stimulation test and the protracted history of seizure activity. The aetiology of the seizures in our cat thus remained unclear. Although idiopathic epilepsy in terms of unknown aetiology remains a possibility, the presence of structural malformations of the brain, even if not related to the supratentorial brain, makes the diagnosis of epilepsy of unknown aetiology debatable. Cerebellar seizures have been reported in people;^16^ however, there is no current evidence that natural cerebellar seizures occur in animals. Nevertheless, experimental studies in cats have demonstrated an inhibitory action of the cerebellum on epileptic seizures.^17^ Therefore, it is possible that a major dysfunctional cerebellum, as in this cat, could lower the forebrain threshold for seizure activity.

DWM is believed to be the result of dysgenesis of the roof of the rhombencephalon at the level of the anterior membranous area.^6^ In humans, several genetic aetiologies have been reported, mainly affecting neuronal division and migration during the development of the cerebellar vermis.^18^ In veterinary medicine, the only reported genetic origin of DWLM is a mutation in the gene *VLDLR* in Eurasier dogs with an autosomal recessive mode of inheritance.^5,19^ The aetiology of the DWLM in our cat is unknown. No information was available about its siblings or parents as the cat was a rescue. However, >3 years of follow-up has suggested a static nature of the disease, most likely congenital or genetic as described in dogs and humans.

Intra-utero feline panleukopenia virus infection has been reported to cause granuloprival cerebellar hypoplasia in cats; however, diagnosis ante mortem is not possible. With feline panleukopenia virus infection, the whole cerebellum is grossly hypoplastic and on MRI there is a characteristic increased CSF space around the whole cerebellum and between the cerebellar folia, which differs from our case.^20–22^

The cat in the present report also had a misshaped occipital bone. The fetal occipital bone originates from the mesoderm and its development occurs before the development of the cerebellum.^23^ The relationship of this bone malformation with the cerebellar malformation is unclear in this cat; however, a developmental abnormal crosstalk between the developing neuroectoderm and mesoderm may have happened.

Currently there is no aetiological treatment for DMW. Symptomatic treatment is recommended, which, in our epileptic ataxic cat, consisted of antiseizure medication and environmental modification at home. In humans, the most deleterious complication of DWM is the dilation of the fourth ventricle and its progression into hypertensive hydrocephalus in up to 80% of the cases. In these cases, symptomatic treatment addresses the secondary CSF accumulation using surgical interventions, such as ventriculoperitoneal shunting.^24^ DWM complications, such as obstructive hydrocephalus, have not been proven in animals. In the largest DWLM case series published, some dogs with DWLM plus seizures also had internal hydrocephalus. However, it is unclear if this is a concomitant unrelated malformation or if it is a DWLM complication.^5^ Most animals with severe DWLM are euthanased, thus it is unknown if complications occur. In our cat, a follow-up of >3 years has shown no clinical progression or complications besides the seizures, which have been static in frequency and phenotype.

The prognosis in people with DWM is variable, with few patients surviving more than 3 years. A good prognostic factor appears to be the presence of normal vermian lobulation in the absence of supratentorial abnormalities.^25,26^ In our case, no concomitant supratentorial malformations were detected and the rostral vermis appeared present with normal lobulation. These may have contributed to the favourable prognosis of our cat. The only DWLM study in which the animals have not been immediately euthanased has shown a good prognosis with static disease. In that study, most of the dogs were alive at the time of publication, with one dog being euthanased due to uncontrolled seizures.^5^ Our cat at the time of publication is 5 years old, presents static neurological status, static seizure frequency and has a good quality of life as perceived by the owner.

## Conclusions

We report a case of DWLM in an adult cat presenting with seizures, its MRI features and long-term outcome. MRI is necessary to diagnose DWM/DWLM and is important to exclude other malformations, especially in the presence of atypical neurological signs, such as seizures. In this case, DWLM carries a good prognosis with the appropriate management of seizures and environmental modifications.
